# Synovial Sarcoma of Ethmoidal Sinus

**DOI:** 10.1055/s-0041-1731634

**Published:** 2021-08-03

**Authors:** Sapna Dhiman, Sarita Negi, Sandeep Moudgil, Jagdeep S. Thakur, Ramesh K. Azad

**Affiliations:** 1 Department of Otolaryngology-Head and Neck Surgery (ENT), Indira Gandhi Medical College, Shimla, Himachal Pradesh, India; 2 Department of Neuro-Radiology and Intervention, Indira Gandhi Medical College, Shimla, Himachal Pradesh, India

**Keywords:** nose, ethmoid sinus, synovial sarcoma, tumor

## Abstract

**Background** Synovial sarcoma is an aggressive soft tissue cancer of extremities mainly and rare in head and neck region, whereas rarest in ethmoidal sinus as only three cases have been reported till date.

**Case Reports** We managed two cases of synovial sarcoma who presented with nasal obstruction, epistaxis, and swelling around the nasofacial region. Endoscopic nasal biopsy and immunohistochemistry markers confirmed synovial sarcoma in both the cases. While one case was managed by surgery and chemoradiation, the second patient received two cycles of ifosfamide-based chemotherapy and succumbed after 6 weeks of diagnosis.

**Conclusion** Head and neck sarcomas are aggressive and carry a poor prognosis. Surgical resection with postoperative radiotherapy is the standard treatment. However, they have a high risk of recurrence and hence aggressive management and close follow-up is warranted for the optimal outcome.

Sarcoma is a soft tissue tumor and contributes less than 1% of total cancer in adults. 1 Most commonly, it occurs in para-articular areas of extremities, closely associated with tendon sheath, bursae, and joint capsule. In less than 5 to 10%, it manifests in head and neck area, but ethmoid sinus is the rarest site of origin as only three cases have been reported in the English literature (PubMed, Scopus, and Google Scholar) till now. 2 3 4 Here, we present two more cases of synovial sarcoma arising in the ethmoid sinus.

## Case 1

**Fig. 1:**
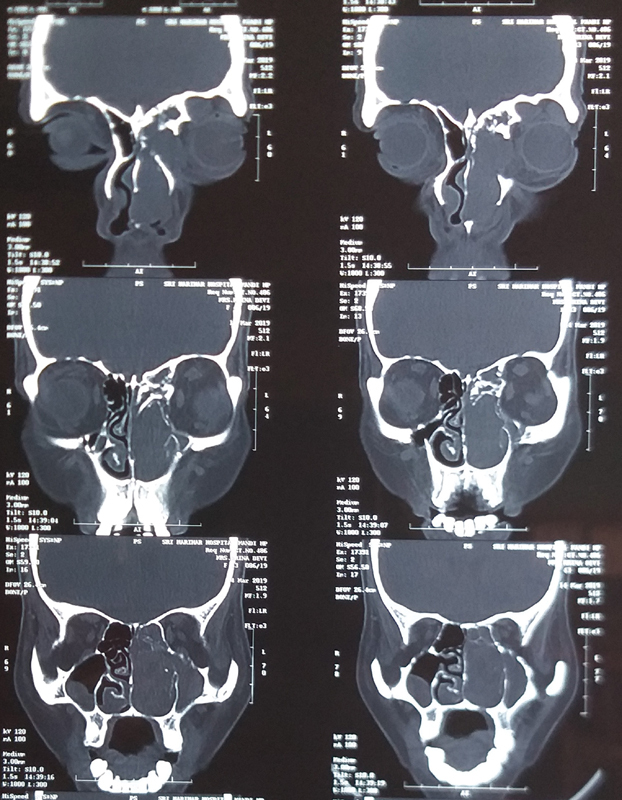
Computed tomography scan showing isodense expansile mass in left nasal cavity, ethmoids, frontal sinus, and orbit.

**Fig. 2:**
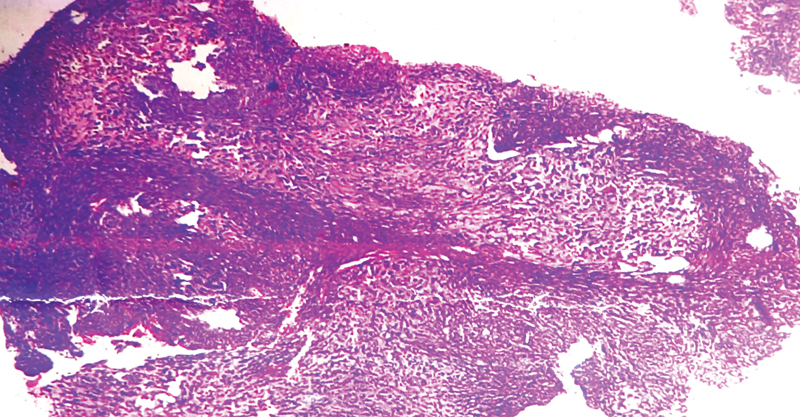
Microphotograph of tumor showing long fascicles of spindle cells with indistinct borders elongated nuclei with fine chromatin with inconspicuous nucleoli (hematoxylin and eosin ×10).

**Fig. 3:**
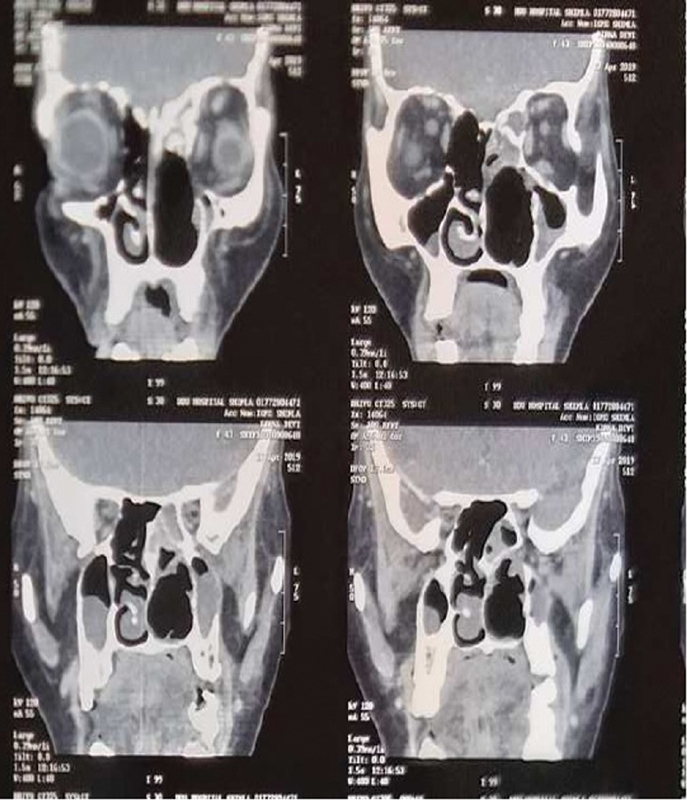
Postoperative computed tomography scan with no evidence of tumor grossly.

In March 2019, a 43-year-old, Asian (Indian) woman presented with progressive nasal obstruction and swelling in the medial canthal region on the left side for the last 4 months. She had occasional epistaxis from the left nostril. She was a high school educated housewife, with no history of any comorbidity, medication, or surgery. The examination found diffuse, soft-firm, nontender swelling in the left supraorbital and brow region. Anterior rhinoscopy found grayish white soft-firm polypoidal mass filling the left nasal cavity. The ophthalmic examination found normal vision and eyeball movements. A probable diagnosis of the nasal polyp with frontoethmoidal mucocele was kept, and she was investigated to confirm the diagnosis. High-resolution computed tomography (CT) ( Fig. 1 ) showed a dense expansile lesion in the ethmoid, sphenoid, and frontal sinus. It was extending to the extraconal compartment of the left orbit. These features were suggestive of a malignant tumor and hence endoscopic biopsy was done. Histopathological examination ( Fig. 2 ) revealed tissue composed of long fascicles of spindle cells with indistinct borders elongated nuclei with fine chromatin with inconspicuous nucleoli. Few focal areas had epithelioid cells morphology and hyalinization. A diagnosis of synovial sarcoma was made, and it was positive for CD-99, TLE100, SMA, and Ki-67 markers. Endoscopic resection of the tumor was performed. The tumor was arising in the ethmoid sinus and involving the anterior part of the middle turbinate and posterior part of the inferior turbinate. It was eroding lamina papyracea and the ascending process of the maxilla. Frontal, maxillary, and sphenoid sinuses had retained secretion. The resection leads to partial medial maxillectomy, complete ethmoidectomy except for cribriform plate/anterior cranial fossa resection, and partial middle turbinectomy. Resection histopathology further confirmed synovial sarcoma; however, tumor-free resection margin could not be confirmed due to microdebrider-assisted surgery and resections close to the important structures such as dura. Postoperative contrast-enhanced CT and magnetic resonance imaging after 3 weeks showed inflammation with no gross residual tumor. In absence of tumor-free resection margin and high-grade sarcoma, she was started with ifosfamide-based chemotherapy and external beam radiotherapy of 60 to 65 Gy; however, 6 months later, she had a swelling in the left medial canthal region, which was a tumor recurrence in the left orbit on radiopathological evaluation ( Fig. 3 ). Salvage surgery in terms of left anterior cranial fossa clearance and orbital exenteration was performed. The patient remained asymptomatic for 1 year, but later, she was lost to follow-up.

## Case 2

In July 2020, an 82-year-old Asian (Indian) woman presented with right nostril obstruction, epistaxis, external nasal deformity associated with swelling of the right upper eyelid, double vision, and headache. The patient had a known case of hypertension and diabetes for the past 5 years. She was recently diagnosed with chronic kidney disease.

On examination, a diffused swelling on the dorsum of the nose and extending to the right medial canthus was observed. Anterior rhinoscopy revealed a grayish white polypoidal mass in the right nasal cavity. On posterior rhinoscopy, there was a polypoidal mass in the nasopharynx on the right side. An ophthalmic examination found a decreased movement of the right eye in superior and medial direction with the absence of corneal reflex. Type “B” tympanogram was also obtained for the right ear. The neck examination was normal. Contrast-enhanced CT of the nose and paranasal sinus revealed the presence of a heterogeneous soft tissue density of size 2.45 × 7.21 × 4.24 cm in the right nasal cavity with superior extension to the right ethmoidal sinus. Its superolateral extension in the extraconal compartment of the right orbit measured 2.66 × 3.72 × 2.65 cm. It caused erosion of the perpendicular plate of the ethmoid bone and lamina papyracea and extended up to the right frontoethmoidal recess. The entire right maxillary sinus and sphenoid sinus were opacified with retained secretions.

**Fig. 4:**
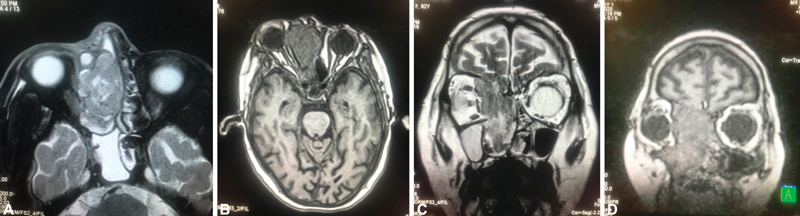
( **A–D** ) Magnetic resonance imaging of case 2 showing tumor primarily in the nasal cavity and ethmoid gallery with intracranial extension.

A magnetic resonance imaging ( Fig. 4A–D ) revealed the presence of an ill-defined altered signal intensity lesion of 6.3 × 2.4 × 6 cm in the right nasal cavity which was heterogeneously hypo-intense on T2/fluid-attenuated inversion recovery (FLAIR). Superiorly, it was extending into the ethmoidal sinus leading to erosion of the cribriform plate and extending intracranially into the anterior cranial fossa. However, no parenchymal involvement was observed. The right lamina papyracea was destructed and the superior oblique and medial rectus were displaced and effaced, displacing the globe anteriorly. Posteriorly, it was extended up to the nasopharynx. T2/FLAIR hyperintensities in the bilateral maxillary sinus and sphenoid sinus were observed due to retained secretions. No evidence of distant metastases was found.

Histopathological examination of the endoscopic biopsy revealed fragments of epithelial tumor component comprising medium-sized tumor cells arranged in sheets and trabeculae with conspicuous peritheliomatous arrangement. At the cytological level, the tumor cell showed a high N:C ratio, round to oval pleomorphic nuclei, fine to coarse granular chromatin, variable conspicuous nucleoli, and scant to the variable eosinophilic cytoplasm. Numerous mitotic and apoptotic fragments were also observed. The tumor was immunoreactive to TLE-1, SMD, BL-2, CD-56, CD-99, SOX-10, S-100, and nonimmunoreactivity to CK, PG3, EMA, synaptophysin, CD-34, desmin, myogenin, HMB-4S, melan-A, and chromogranin-A. All these features were confirmatory for the synovial sarcoma. Although surgery is the treatment of choice for synovial sarcoma, a multidisciplinary board decided chemoradiation based on advanced stage and age of the patient, and ifosfamide-based chemotherapy was started. The patient received two cycles of chemotherapy, but died after 6 weeks of diagnosis.

## Discussion

The synovial sarcoma originates in the periarticular areas of the extremities in the second and third decades with a male predilection. Hereditary retinoblastoma, neuroblastoma, and Lynch syndrome are a predisposing factor for the development of sarcomas. 5 It contributes 8 to 10% in soft tissue sarcomas, whereas out of these, 5 to 10% occur in the head and neck region. 2 6 It has been found in almost all locations of the head and neck region, but its occurrence in the paranasal sinus is rare. 5 7 8 Further, ethmoidal sinus synovial sarcoma is rarer as only three cases has been reported till date.

Synovial sarcoma is a high-grade sarcoma and usually remains asymptomatic until compressive symptoms start appearing in adjoining areas similar to our case. It may present with nasal obstruction, pain, earache, sore throat, epistaxis, and rarely diplopia or vision loss. 5

A CT scan can indicate the malignant nature of the nasal mass, but an endoscopic biopsy is confirmatory for this rare tumor. It is treated by surgery followed by chemoradiation. 9 Basic principle of oncological surgery of resection with negative margins (R0) should be the aim, but it is difficult to achieve in the paranasal sinus surgery, especially endoscopic sinus surgery due to important structures close to the tumor and microdebrider/piecemeal resection. Moreover, endoscopic surgery requires expertise in such cases, and hence, an open surgical approach is helpful and advantageous especially in advance cases. Frozen section after complete resection of the tumor will assure complete resection in both approaches. Despite this, it has high recurrence rates usually within 2 years. 10 11 12 This recurrence is significantly quite high in skull base and paranasal sinus synovial sarcomas. 13 It depends on grade, stage (bone invasion, size), and site. 14 Regular endoscopic and radiological examination should be performed during follow-up period. A positron emission CT has advantages over other radiological investigation in detecting recurrence who otherwise missed, but should be advised after 12 weeks of surgery to avoid false-positive results.

Postoperative or definitive radiotherapy proved to be beneficial in sarcomas. 9 Andrä et al reviewed 26 cases of head and neck sarcomas with median age of 64 years and male predominance. High-grade lesions predominantly angiosarcoma, malignant fibrous histiocytoma, and synovial sarcoma were found in 25 cases. Various anatomical structures involved were skull (including skin), paranasal sinus/orbit, and neck (including pharynx/larynx) with median tumor size of 4.6 cm. Surgical excision was performed in 81% cases resulting in tumor-free margins in 38% cases only, while 23% had positive margins and gross residual disease remained in 19% cases. All cases were treated with 66 Gy postoperative or definitive radiotherapy, while half of them also received sequential chemotherapy. They found 5-year local control of 86%, while overall survival was 82%. 9 Synovial sarcomas are chemosensitive and chemoradiation increases the prognosis, but overall survival remains poor in high-grade sarcomas including synovial sarcoma. 9

## Conclusion

Recent experimental studies and clinical trials on targeted therapy have provided promising results in recurrent synovial sarcomas. 15 16 The U.S. Food and Drug Administration has approved pazopanib, a multikinase inhibitor for advanced synovial sarcomas not responding to anthracycline-based chemotherapy. 17 However, despite these advances, the 10-year survival of metastatic synovial sarcoma is still 8.9% compared with 69% in localized sarcoma. 18 Most metastases originate from hematogenous dissemination, although up to 20% spread through the lymphatic to regional lymph nodes. These two cases emphasize the rarity and poor prognosis of the head and neck sarcoma especially the synovial sarcoma, despite the availability of various treatment modalities.

## References

[1] Jemal A Siegel R Xu J Ward E Cancer statistics, 2010 CA Cancer J Clin 2010 60 05 277 300 20610543 10.3322/caac.20073

[2] Kraus D H Dubner S Harrison L B Prognostic factors for recurrence and survival in head and neck soft tissue sarcomas Cancer 1994 74 02 697 702 8033050 10.1002/1097-0142(19940715)74:2<697::aid-cncr2820740224>3.0.co;2-a

[3] Jain A Saxena A Meher R Khurana N Synovial sarcoma of the ethmoid sinus Eur Ann Otorhinolaryngol Head Neck Dis 2018 135 06 453 455 30352776 10.1016/j.anorl.2017.10.007

[4] Wong H T Ho C Y Nazarina A R Prepageran N Synovial sarcoma of the ethmoidal sinus J Laryngol Otol 2014 128 11 1022 1023 25274107 10.1017/S0022215114002151

[5] Salcedo-Hernández R A Lino-Silva L S Luna-Ortiz K Maxillary sinus sarcoma: epidemiological and clinicopathological experience of 25 years in a national reference cancer centre Indian J Otolaryngol Head Neck Surg 2014 66 04 359 364 10.1007/s12070-012-0522-9 PMC4571464 26396944

[6] Kransdorf M J Malignant soft-tissue tumors in a large referral population: distribution of diagnoses by age, sex, and location AJR Am J Roentgenol 1995 164 01 129 134 7998525 10.2214/ajr.164.1.7998525

[7] Bukachevsky R P Pincus R L Shechtman F G Sarti E Chodosh P Synovial sarcoma of the head and neck Head Neck 1992 14 01 44 48 1320596 10.1002/hed.2880140110

[8] Mallen-St Clair J Arshi A Abemayor E St John M Factors associated with survival in patients with synovial cell sarcoma of the head and neck: an analysis of 167 cases using the SEER (Surveillance, Epidemiology, and End Results) database JAMA Otolaryngol Head Neck Surg 2016 142 06 576 583 27100936 10.1001/jamaoto.2016.0384 PMC6173585

[9] Andrä C Rauch J Li M Excellent local control and survival after postoperative or definitive radiation therapy for sarcomas of the head and neck Radiat Oncol 2015 10 140 26156022 10.1186/s13014-015-0449-x PMC4496934

[10] Hoffman H T Robinson R A Spiess J L Buatti J Update in management of head and neck sarcoma Curr Opin Oncol 2004 16 04 333 341 15187888 10.1097/01.cco.0000127880.69877.75

[11] Colville R J Charlton F Kelly C G Nicoll J J McLean N R Multidisciplinary management of head and neck sarcomas Head Neck 2005 27 09 814 824 16086411 10.1002/hed.20232

[12] Nielsen T O Poulin N M Ladanyi M Synovial sarcoma: recent discoveries as a roadmap to new avenues for therapy Cancer Discov 2015 5 02 124 134 25614489 10.1158/2159-8290.CD-14-1246 PMC4320664

[13] Owosho A A Estilo C L Rosen E B Yom S K Huryn J M Antonescu C R A clinicopathologic study on SS18 fusion positive head and neck synovial sarcomas Oral Oncol 2017 66 46 51 28249647 10.1016/j.oraloncology.2016.12.021 PMC5640264

[14] Harb W J Luna M A Patel S R Ballo M T Roberts D B Sturgis E M Survival in patients with synovial sarcoma of the head and neck: association with tumor location, size, and extension Head Neck 2007 29 08 731 740 17274049 10.1002/hed.20564

[15] Jones S E Fleuren E DG Frankum J ATR is a therapeutic target in synovial sarcoma Cancer Res 2017 77 24 7014 7026 29038346 10.1158/0008-5472.CAN-17-2056 PMC6155488

[16] Dallos M Tap W D D'Angelo S P Current status of engineered T-cell therapy for synovial sarcoma Immunotherapy 2016 8 09 1073 1080 27485079 10.2217/imt-2016-0026 PMC5618931

[17] EORTC Soft Tissue and Bone Sarcoma Group PALETTE study group van der Graaf W T Blay J Y Chawla S P Pazopanib for metastatic soft-tissue sarcoma (PALETTE): a randomised, double-blind, placebo-controlled phase 3 trial Lancet 2012 379(9829):1879 1886 22595799 10.1016/S0140-6736(12)60651-5

[18] Sultan I Rodriguez-Galindo C Saab R Yasir S Casanova M Ferrari A Comparing children and adults with synovial sarcoma in the Surveillance, Epidemiology, and End Results program, 1983 to 2005: an analysis of 1268 patients Cancer 2009 115 15 3537 3547 19514087 10.1002/cncr.24424

